# Small molecules targeted to the microtubule–Hec1 interaction inhibit cancer cell growth through microtubule stabilization

**DOI:** 10.1038/onc.2017.320

**Published:** 2017-09-18

**Authors:** M Ferrara, G Sessa, M Fiore, F Bernard, I A Asteriti, E Cundari, G Colotti, S Ferla, M Desideri, S Buglioni, D Trisciuoglio, D Del Bufalo, A Brancale, F Degrassi

**Affiliations:** 1 Institute of Molecular Biology and Pathology, CNR National Research Council, Rome, Italy; 2 School of Pharmacy & Pharmaceutical Sciences, Cardiff University, Cardiff, UK; 3 Advanced Diagnostics and Technological Innovation Department, Regina Elena National Cancer Institute, Rome, Italy

## Abstract

Highly expressed in cancer protein 1 (Hec1) is a subunit of the kinetochore (KT)-associated Ndc80 complex, which ensures proper segregation of sister chromatids at mitosis by mediating the interaction between KTs and microtubules (MTs). HEC1 mRNA and protein are highly expressed in many malignancies as part of a signature of chromosome instability. These properties render Hec1 a promising molecular target for developing therapeutic drugs that exert their anticancer activities by producing massive chromosome aneuploidy. A virtual screening study aimed at identifying small molecules able to bind at the Hec1–MT interaction domain identified one positive hit compound and two analogs of the hit with high cytotoxic, pro-apoptotic and anti-mitotic activities. The most cytotoxic analog (SM15) was shown to produce chromosome segregation defects in cancer cells by inhibiting the correction of erroneous KT–MT interactions. Live cell imaging of treated cells demonstrated that mitotic arrest and segregation abnormalities lead to cell death through mitotic catastrophe and that cell death occurred also from interphase. Importantly, SM15 was shown to be more effective in inducing apoptotic cell death in cancer cells as compared to normal ones and effectively reduced tumor growth in a mouse xenograft model. Mechanistically, cold-induced MT depolymerization experiments demonstrated a hyper-stabilization of both mitotic and interphase MTs. Molecular dynamics simulations corroborate this finding by showing that SM15 can bind the MT surface independently from Hec1 and acts as a stabilizer of both MTs and KT–MT interactions. Overall, our studies represent a clear proof of principle that MT-Hec1-interacting compounds may represent novel powerful anticancer agents.

## Introduction

Aneuploidy and chromosome instability are strongly involved in tumorigenesis. However, studies on the cancer susceptibility of mouse models heterozygous for genes controlling chromosome segregation suggest that aneuploidy may also act as a tumor suppressive mechanism, depending on the tissue analyzed and its intrinsic chromosome instability.^1, 2^ This evidence suggests that excessive chromosome mis-segregation may compromise genome stability and be incompatible with cell viability. Concordantly, partial downregulation of mitotic checkpoint proteins in tumor cell lines produced cancer cell death associated with severe chromosome mis-segregation.^3^ Moreover, overexpression of cyclin E inducing multipolar divisions and numerous chromosome segregation errors was found to have a significant anti-tumorigenic effect in a mouse model for lung cancer.^4^ Consequently, the idea of promoting cell death by inducing massive chromosome mis-segregation at mitotic division was proposed as a therapeutic strategy to selectively eliminate actively proliferating tumor cells.^5^

Highly expressed in cancer protein 1 (Hec1) is a subunit of the kinetochore (KT)-associated Ndc80 complex, which mediates the attachment of microtubules (MTs) to the KT during mitotic division and ensures proper alignment and segregation of sister chromatids.^6, 7^ Moreover, HEC1 mRNA is highly expressed in many malignancies and elevated Hec1 expression is associated with negative prognosis in multiple cancer types.^8, 9, 10^ Therefore, Hec1 represents a promising molecular target for developing new therapeutic drugs that exert their anticancer property by producing massive chromosome aneuploidy and cell death in cancer cells. We previously showed that expression of a Hec1 protein modified at its N-terminus, the region of interaction with MTs, massively kills cancer cells both *in vitro* and in tumor xenografts. Live cell imaging of N-terminus modified Hec1-expressing cells demonstrated that cancer cell death is triggered by a prolonged mitotic arrest owing to an attempted chromosome segregation within multipolar spindles followed by cell death at mitosis.^11, 12^ These studies provided a clear proof of concept that the KT protein Hec1 can be a good target for developing anticancer therapies.

X-ray crystallographic studies have revealed that the N-terminal globular head of the Hec1 protein directly binds the MT lattice through a calponin homology domain.^13, 14^ Structural reconstructions of Ndc80 complexes bound to MTs have further restricted the region of interaction by identifying a region within the calponin homology domain of Hec1 named ‘toe’ region that binds a pocket on the MT present at both intra-dimer and inter-dimer tubulin interfaces.^15, 16^ In the ‘toe’ region positively charged amino acid residues interact with negatively charged residues on the ‘toe print’ MT interface, and residues important for MT binding within the ‘toe’ have also been identified.^14, 17, 18^ The ‘toe’–‘toe print’ interaction interface represents an ideal target for developing new compounds that specifically inhibit/modify the MT-Hec1 interaction and can potentially produce massive chromosome mis-segregation and cancer cell death.

In this study, the SPECS library of commercially available compounds was screened *in silico* aiming to find small molecules (SMs) able to bind at the interaction domain between MTs and Hec1. The adopted approach has led to the identification of a first hit compound and an analog of the initial hit with promising anticancer properties.

## Results

### Virtual screening

**Figure 1:**
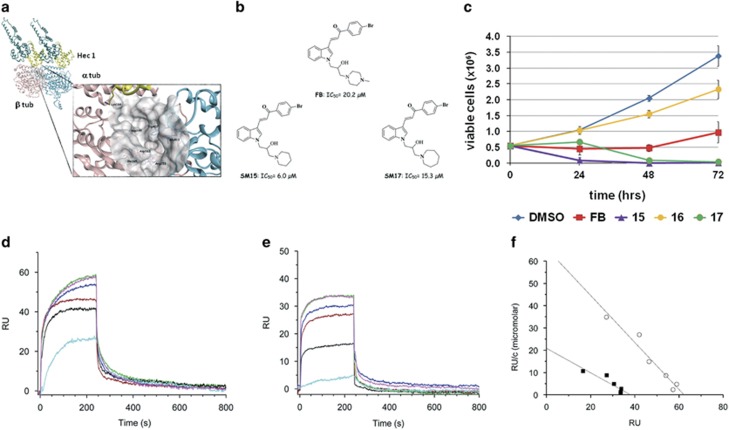
A virtual screening targeted to the MT–Hec1 interaction identifies highly cytotoxic compounds. (**a**) Crystal structure of the α (sky blue) and β (pink) tubulin dimer in association with the Ndc80-SPC25 chimera protein (teal). The ‘toe’ region of Hec1 is in yellow. The zoom highlights the pocket (represented as gray molecular surface) at the interface between the two tubulin monomers where the virtual screening study has been performed. Important amino acid residues from α-tubulin chain and β-tubulin chain are reported. Hec1 Lys166 is in proximity of the selected pocket. The protein backbone is shown as ribbon. (**b**) Chemical structure of FB and its derivatives SM15 and SM17. (**c**) Time course viability assay for selected compounds (FB, SM15, SM16, SM17). Data are means±s.e.m. of 2–4 independent experiments. (**d**) Sensorgrams showing the interaction between MTs, immobilized on a COOH5 chip, and SM15 at different concentrations. (**e**) Sensorgrams showing the interaction between tubulin, immobilized on a COOH5 chip, and SM15 at different concentrations. Concentrations of SM15 were: 0.78 μM: cyan; 1.56 μM: black; 3.12 μM: red; 6.25 μM: blue; 12.5 μM: green; 25 μM: magenta. The increase in RU relative to baseline indicates complex formation; the plateau region represents the steady-state phase of the interaction, whereas the decrease in RU represents dissociation of SM15 from immobilized ligands after injection of running buffer. (**f**) Scatchard plots of SPR experiments showing the interaction (that is, RU values at equilibrium) of SM15 at different concentrations with MTs (circles) and tubulin (squares). Upon linear fitting, KD values of 0.94±0.13 μM and 1.8±0.4 μM were calculated for the SM15 interaction with MTs and tubulin, respectively.

The Hec1 ‘toe’ region was demonstrated to bind the MT wall by interacting with intra-dimer and inter-dimer tubulin interfaces. Examining the model of the Ndc80 kinetochore complex (PDB code 3IZ0), an interesting and well-defined pocket was found at the interface between the two tubulin subunits (Figure 1a). Although this pocket seems not directly involved in the MT–Hec1 interaction, it is adjacent to the tubulin-Hec1 contact area and in proximity of Lys166, which is thought to be part of a MT conformation sensor.^16^ The selected pocket, defined by Trp407 and Glu411 (α-tubulin chain) and Ile165, Asp163, Asp199 and Arg253 (β-tubulin chain), was used to screen the SPECS library of commercially available compounds (~350.000 SMs) aiming at identifying chemical inhibitors/modulators of the MT–Hec1 interaction. A high-throughput virtual screening identified 15 SMs as potential inhibitors/modulators of MT–Hec1 interactions. Among the 15 selected molecules, compound FB induced alterations in the mitotic process and reduced cell viability in HeLa cells (Supplementary Figure 1, IC_50_=20.18 μM). To explore the structure-activity relationship and possibly increase the affinity for the target and the biological potency, several FB analogs were selected and their IC_50_ was evaluated in dose–response experiments in HeLa cells (Supplementary Figure 2). Among the FB analogs, two compounds more cytotoxic than the lead compound (SM15 and SM17: IC_50_=6.0 μM and 15.3 μM, respectively) and one inactive compound (SM16: IC_50_>100 μM) were identified. The structures of FB and its active analogs are reported in Figure 1b. Time course experiments confirmed IC_50_ studies by showing the high potency of SM15 and SM17 in inhibiting cell proliferation (Figure 1c). Cancer specificity of the most cytotoxic molecule SM15 was then investigated by extending the analysis to a non tumorigenic mammary epithelial cell line (MCF10A cells) and to breast cancer MDA-MB-231 cells. HeLa and MDA-MB-231 cells were found more sensitive than MCF10A cells to the growth inhibitory and cell killing effects of SM15 in the dose range of 1–30 μM (Supplementary Figure 3), demonstrating that SM15 is a stronger inducer of cell death in cancer cells as compared with normal ones.

To support the assumption that the analogs directly interact with MTs, SM15 was subjected to surface plasmon resonance (SPR), which allows the evaluation of kinetics and affinity of the binding between proteins and SMs. SPR experiments showed that SM15 interacts directly and with high affinity with both assembled MTs (Figure 1d) and dimeric tubulin (Figure 1e). Scatchard plots of SPR experiments showed that SM15 binds to MTs with a higher affinity with respect to tubulin dimers (*K*_D_=0.94±0.1 μm and 1.80±0.4 μM, respectively; Figure 1f).

### Cell cycle arrest and apoptosis

**Figure 2:**
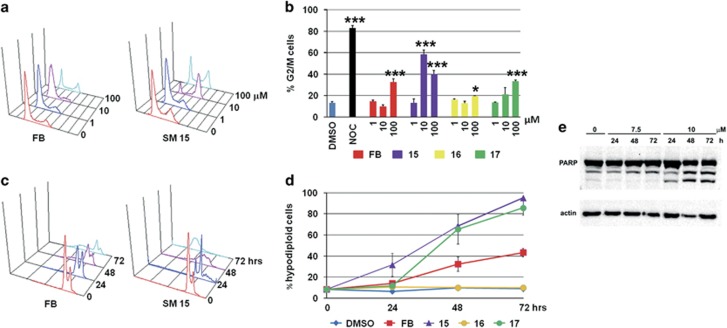
MT-Hec1-interacting SMs lead to a G2/M phase accumulation and induce apoptosis in HeLa cells. (**a**) Representative flow cytometric histograms of cell cycle distribution following 24 h exposure to different concentrations of FB and SM15. *X* axis=DNA content (linear scale), *Y* axis=number of events. (**b**) Quantitative analysis of the percentage of cells in G2/M phases of the cell cycle after 24 h exposure to FB, SM15, SM16 and SM17. Data are means±s.e.m. of 2–4 independent experiments. 1 μM nocodazole (NOC) was used as positive control. ***P < 0.001. (**c**) Representative flow cytometric histograms of the hypodiploid peak following different exposure times to 10 μM FB or SM15. *X* axis=DNA content (log scale), *Y* axis=number of events. (**d**) Quantitative analysis of the percentage of hypodiploid cells following different exposure times to 10 μM FB, SM15, SM16 and SM17. Data are means±s.e.m. of 2–4 independent experiments. (**e**) Western blotting analysis of PARP cleavage after different exposure times to 7.5 and 10 μM SM15. Actin is shown as loading control.

To characterize the cell death pathway activated by the SMs we evaluated cell cycle distribution in HeLa cells by flow cytometry. SM15 treatment arrested HeLa cells in the G2/M phases of the cell cycle more efficiently than FB (Figures 2a and b) and significantly enhanced the percentage of hypodiploid apoptotic cells (Figures 2c and d). A strong accumulation of cells in the G2/M phases was associated with an increment in polyploid cells for 24 h treatments. This was followed by a massive induction of hypodiploid cells at later times (Figures 2b and d and Supplementary Figure 4). These findings confirmed the cytotoxic potential of both analogs and suggested that cell death was dependent on the G2/M arrest, presumably a mitotic arrest. Induction of apoptosis was confirmed by the cleavage of the apoptotic marker poly ADP-ribose polymerase, by caspase 3 activation and by the reduction in DNA fragmentation observed in the presence of the pan-caspase inhibitor Z-VAD-fmk in both HeLa and MDA-MB-231 cells (Figure 2e, Supplementary Figure 5).

### Analysis of mitosis

**Figure 3:**
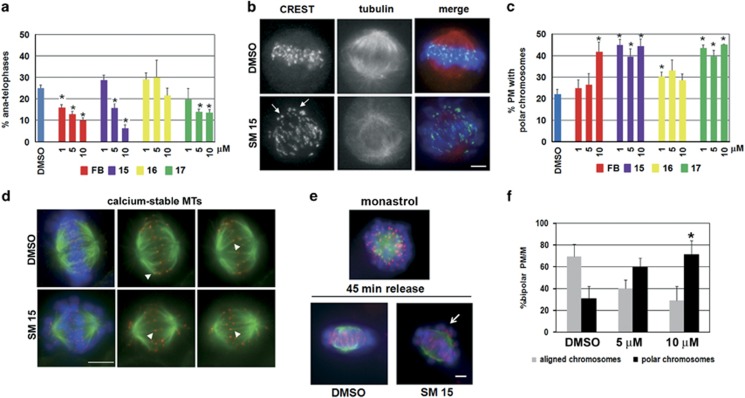
MT-Hec1-interacting SMs disrupt mitotic division in HeLa cells. (**a**) Quantitative analysis of anaphase percentage after 3 h treatment with FB, SM15, SM16 and SM17. Data are means±s.e.m. obtained by scoring 240 mitoses per condition in three independent experiments. (**b**) Representative images of untreated cells showing chromosomes aligned to the metaphase plate and SM15-treated cells with chromosomes remaining at the spindle poles, as identified by KT signals at spindle poles (arrows). Mitotic spindles (red) and KTs (green) are visualized by α-tubulin and CREST antibodies. Chromosomes are identified by DAPI staining (blue). (**c**) Quantitative analysis of prometaphases (PM) showing polar chromosomes following 3 h exposure to FB, SM15, SM16 and SM17. Data are means±s.e.m. obtained by scoring 240 PM per condition in three independent experiments. (**d**) Representative images of calcium-resistant MTs in HeLa cells treated with DMSO or 10 μM SM15 for 3 h. MTs (green) and KTs (red) are visualized by immunostaining α-tubulin and Hec1. Chromosomes are identified by DAPI staining (blue). Images in the first column are maximum intensity projections of a Z series of optical sections at 0.5 μm interval. In the second and third column single optical sections of MTs and KTs are presented to better visualize KT–MT attachments (arrowheads). (**e**) Representative images of MON-induced monopolar spindles (monastrol), fully aligned bipolar spindles in control cells (DMSO) or bipolar spindles with polar chromosomes in SM15-treated cells (SM15) at the end of the recovery time from MON arrest (45 min release). Cells were incubated with 100 μM MON for 4 h and then released in 10 μM MG-132-containing medium with or without 10 μM SM15. (**f**) Quantitative analysis of prometaphases/metaphases (PM/M) showing aligned chromosomes or polar chromosomes after 45 min recovery time from MON arrest in DMSO-treated and SM15-treated cells. Data are means±s.e.m. obtained by scoring ⩾100 PM/M per condition in two independent experiments. *P<0.05. Bars=5 μm.

Next, we sought to further investigate the possible mechanisms responsible for the observed cytotoxic effects. As cells defective for Hec1 function show chromosome segregation and spindle abnormalities,^6, 17, 18, 19^ we analyzed the mitotic phenotypes of SM-treated HeLa cells. Mitotic progression from metaphase to anaphase was clearly inhibited after 3 h exposure to SM15, SM17 or FB (Figure 3a), suggesting activation of the spindle assembly checkpoint. Unattached or faulty attached KTs activate spindle assembly checkpoint that delays mitotic progression into anaphase until sister KTs attach properly to spindle MTs and chromosomes congress to the metaphase plate.^20, 21^ We then analyzed chromosome congression by immunostaining with antibodies that visualize KTs and mitotic spindles (Figure 3b). Treatment with SM15 and SM17 significantly increased the frequencies of unaligned, polar KTs, indicative of unattached or syntelically attached chromosomes (Figure 3b, SM15, Figure 3c).^22^ These findings suggest that SMs delay mitotic progression by interfering with KT–MT attachment, thereby activating spindle assembly checkpoint. Consistently, KTs of unaligned chromosomes in SM15-treated cells accumulated the checkpoint protein BubR1 (Supplementary Figure 6). To test the nature of KT–MT interactions in the presence of MT-Hec1-interacting SM15, we took advantage of a calcium buffer that selectively depolymerizes both non-KT MTs and unstable KT–MT interactions.^23^ Incubation with calcium buffer before fixation demonstrated that interactions between Hec1 and MTs, visualized by antibody staining, were maintained in the presence of SM15 (Figure 3d, arrowheads), indicating that the compound does not act as classical MT depolymerizing agents, such as nocodazole or colchicine, that prevent the formation of stable KT–MT interactions. The occurrence of polar chromosomes in the presence of stable KT–MT interactions led to the hypothesis that SM15 could inhibit the correction of syntelic or monotelic KT–MT attachments that maintain chromosomes close to the spindle poles.^22^ To test this hypothesis, we used monastrol (MON), a chemical inhibitor of the kinesin-5 protein Eg5. As Eg5 activity is required for spindle pole separation at mitosis, MON-induced Eg5 inhibition produces monopolar spindles with sister KTs syntelically or monotelically attached to the single spindle pole. Upon MON removal, monopoles reorganize to form bipolar spindles and incorrect attachments are corrected (Figure 3e).^22, 24^ In this assay, SM15 strongly inhibited the correction of erroneous attachments as shown by the persistence of polar chromosomes after release from MON arrest (Figures 3e and f). Altogether, these findings suggest that SM15 inhibits KT–MT error correction possibly by stabilizing KT–MT interactions.

### Live cell imaging

**Figure 4:**
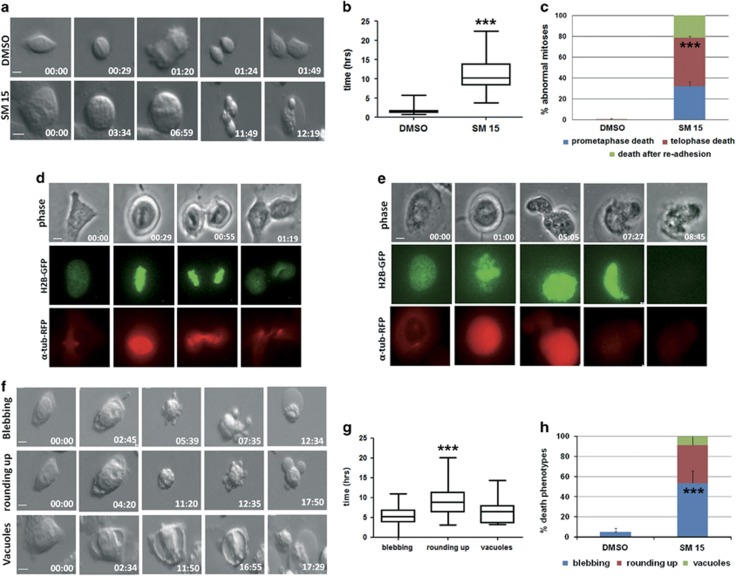
Apoptotic cell death intervenes from mitosis and interphase after treatment with MT-Hec1-interacting SM15. (**a**) Still images of an untreated HeLa cell (DMSO) or a SM15- treated HeLa cell (SM15) recorded by time-lapse microscopy under differential interference contrast (DIC). Time is given in h:min. Cells enter mitosis at time 00.00. (**b**) Box-plots with whiskers showing minimum and maximum values of the time spent in mitosis. (**c**) Quantitative analysis of cell death phenotypes of mitotic HeLa cells recorded as in **a**. Data are means±s.e.m. of two experiments. DMSO *N*=50; SM15 *N*=28. *** *P*<0.001 comparing the mean value for death in telophase in control vs SM15-treated samples. (**d**) An untreated H2B-GFP and α-tubulin-RFP expressing U2OS cell recorded by time-lapse microscopy under fluorescence and phase contrast. Chromosome congression and spindle formation are visualized by H2B-GFP and α-tubulin-RFP. (**e**) An H2B-GFP and α-tubulin-RFP expressing U2OS cell treated with SM15 and recorded as in **d**. Chromosomes persist scattered at the poles (01:00) and then collapse (05:05) until DNA diffuses from the cell when the death process is completed (08:45). (**f**) Still images of interphase HeLa cells recorded as in (**a**) undergoing apoptotic cell death after treatment with SM15. Cells round up and partially detach from the substrate at time 00.00. (**g**) Box-plots with whiskers showing minimum and maximum values of the time spent to complete the death process in HeLa cells. ***P<0.001 comparing mean time in rounding up vs blebbing samples. (**h**) Quantitative analysis of cell death phenotypes in interphase HeLa cells. DMSO *N*=12; SM15 *N*=93. ***P<0.001 comparing the mean value for blebbing in control vs SM15-treated samples. Bars=5 μm.

To assess the fate of mitoses with defective KT–MT interactions, we followed mitotic division by time-lapse microscopy in HeLa cells (Figure 4). Whereas untreated cells progressed through mitosis and reattached to the substrate within a time interval of ~1 h, cells entering mitosis in the presence of SM15 experienced a marked lengthening of mitosis with a mean mitotic time exceeding 11 h (Figures 4a and b; Supplementary material, video 1 and 2). All mitotic cells died during mitosis or shortly after reattachment to the substrate. Visual inspection of the videos demonstrated that cell death occurred in prometaphase or intervened after chromosome segregation, or nuclear blebbing and cell death occurred after cell re-adhesion (Figures 4a and c). Visualization of H2B-GFP and α-tubulin-RFP fluorescence in U2OS osteosarcoma cells allowed us to follow the dynamics of chromosome congression and mitotic spindle organization in untreated live cells (Figure 4d, Supplementary material, video 3) or cells treated with SM15 (Figure 4e, Supplementary material, video 4). Consistent with our immunofluorescence results, chromosomes did not properly congress to the metaphase plate in treated cells. Furthermore, prometaphase arrest was followed by plasma membrane movements, DNA collapse and cell death (Figure 4e). The recorded death phenotypes recapitulate the morphological signs of mitotic catastrophe, a specific type of apoptosis elicited by mitotic disruption.^25, 26^ Hence, MT-Hec1-interacting compounds can promote cell death via mitotic catastrophe. Surprisingly, we also detected massive cell death starting from interphase in both HeLa (Figures 4f and h) and U2OS cells (data not shown). This occurred through extensive membrane blebbing (Figure 4f, blebbing) or membrane blebbing preceded by cell contraction (Figure 4f, rounding up) or formation of large vacuoles (Figure 4f, vacuoles, Supplementary material, video 5), with a time interval from the initial signs of cell contraction to the end of cell movements ranging from 5 to 9 h (Figure 4g). Altogether, these results show that SM15 induces cell death in tumor cell lines both by mitotic catastrophe and by apoptotic cell death directly from interphase.

### Stabilization o f MTs

**Figure 5:**
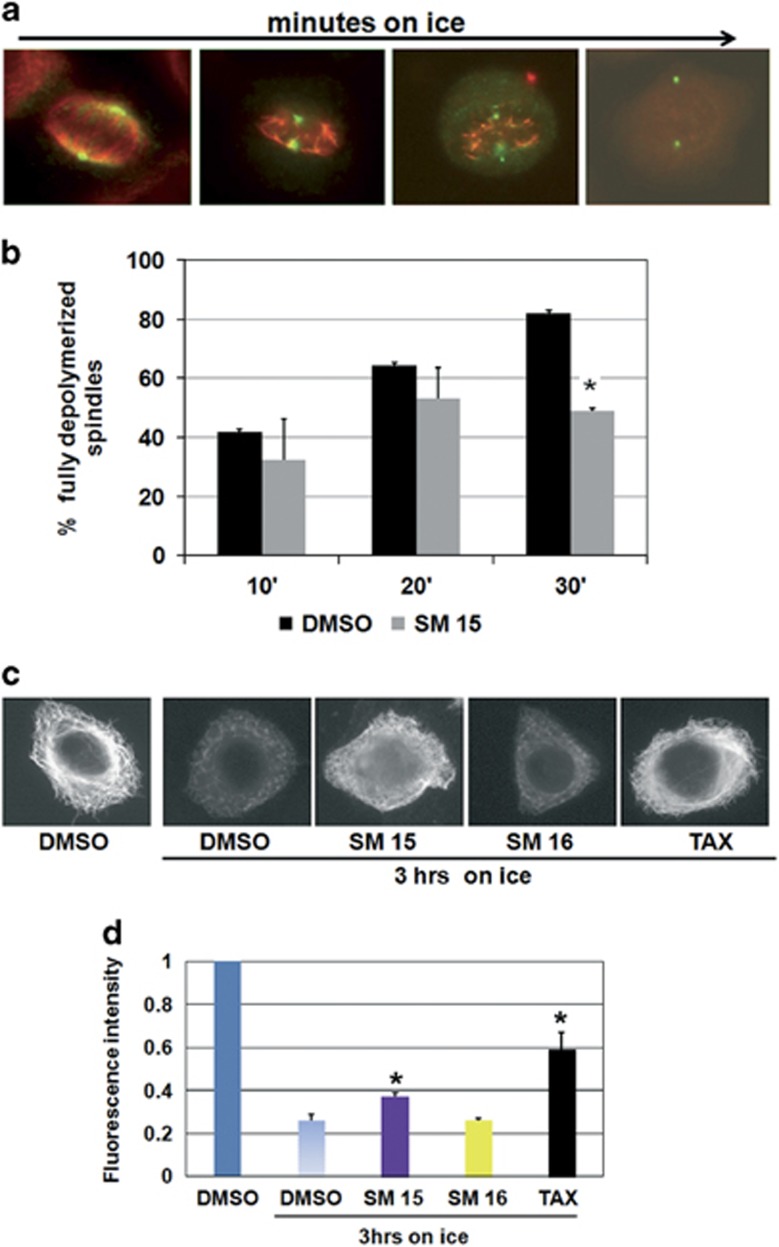
Mitotic and interphase MTs are stabilized by SM15 treatment. (**a**) Representative images of MT depolymerization during incubation on ice. After short incubation times on ice, non-KT MTs depolymerize leaving behind only K-fibers, that is, the bundle of MTs attached to KTs; for longer times on ice also K-fibers depolymerize leading to fully depolymerized spindles (third and forth image in the row). MTs (red) and spindle poles (green) are visualized by α-tubulin and γ-tubulin immunostaining. (**b**) Quantitative analysis of the percentage of fully depolymerized spindles in cells exposed to DMSO or 10 μM SM15 for 3 h prior and during 10, 20 or 30 min incubation on ice. The graph shows means±s.e.m. by scoring 240 mitoses per condition in three independent experiments. (**c**) Representative images of the interphase MT network in the different treatment conditions as visualized by α-tubulin immunostaining. (**d**) Quantitative analysis of interphase MT depolymerization in cells exposed to 1% DMSO, SM15, SM16 (10 μM) or 0.1 μM taxol (TAX) for 3 h prior and during 3 h of incubation on ice. The graph shows means of α-tubulin fluorescence intensity±s.e.m. (in arbitrary units) by measuring 20 cells per condition from two independent experiments. α-tubulin fluorescence intensity in untreated cells is set as 1. *P<0.05.

The unexpected finding that SM15 caused a strong induction of cell death from interphase prompted us to investigate the consequences of SM15 treatment on the MT network as a whole. To this aim, we induced MT depolymerization by incubating cells on ice (Figure 5a) and assessed the resistance of SM-treated cells to this procedure. Our data show that spindle MTs in control cells progressively depolymerized with increasing ice incubation times (Figure 5a) so that >80% of dimethylsulfoxide (DMSO)-treated mitoses were fully depolymerized after 30 min on ice (Figure 5b). At opposite, spindle MTs in SM15-treated cells were resistant to MT depolymerization so that only half of the cells were fully depolymerized at the same time point (Figure 5b). A longer cold exposure (3 h) was then performed to depolymerize interphase MTs that are characterized by a lower dynamic instability.^27^ In line with data on mitotic cells, the depolymerization of the cytoplasmic MT network caused by the low temperature in DMSO-treated cells was recovered in cells treated with SM15 (Figures 5c and d). Altogether, these findings demonstrate that both mitotic and interphase MTs are stabilized by SM15.

### Studies on tumor growth *in vivo*

**Figure 6:**
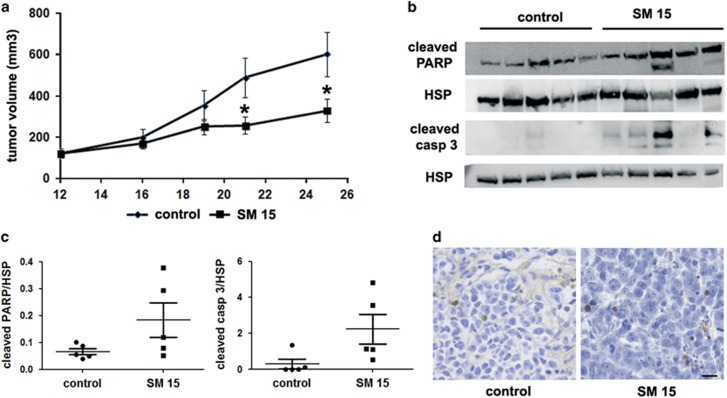
MT-Hec1-interacting SM15 inhibits HeLa tumor growth *in vivo*. (**a**) Tumor growth curves in mice with or without intraperitoneal injection of SM15 (10 mg/kg) for five consecutive days for three weeks starting from tumor palpability. (**b**) Western blotting analysis of cleaved PARP and cleaved caspase 3 in protein lysates from tumors excided at sacrifice. HSP is shown as loading control. (**c**) Cleaved PARP/HSP ratio and cleaved caspase 3/HSP ratio from the densitometric analysis of western blots in **b**. Each scatter plot presents individual values of control and SM15-treated animals. (**d**) Representative images of TUNEL staining in histological sections from tumors excided at sacrifice. Bar=20 μm. *P<0.05 by non-parametric Mann–Whitney test.

Next, we investigated the *in vivo* efficacy of SM15. To this purpose, established HeLa xenografts in nude mice were treated with SM15 starting from tumor palpability. As reported in Figure 6a, *in vivo* experiments confirmed *in vitro* results on HeLa cells. SM15-treated animals showed a significant inhibition of tumor growth starting from day 21 up to the sacrifice of the mice at day 25. Interestingly, apoptosis was confirmed as the death pathway activated by SM15, as determined by the enhanced poly ADP-ribose polymerase and caspase 3 cleavage in tumor protein lysates (Figures 6b and c) and by TUNEL assay (Figure 6d). Indeed, an increase of about two fold in the number of TUNEL positive cells/field was observed in treated xenografts (6.6±1.2 vs 10.2±1.4). Finally, SM15 did not produce any adverse health effects on mice, as monitored by diet consumption, body-weight loss, postural and behavioral changes. Altogether, these findings represent a clear proof of principle that MT-Hec1-interacting SM15 can exert anti-tumor activity.

### Molecular dynamics simulation

**Figure 7:**
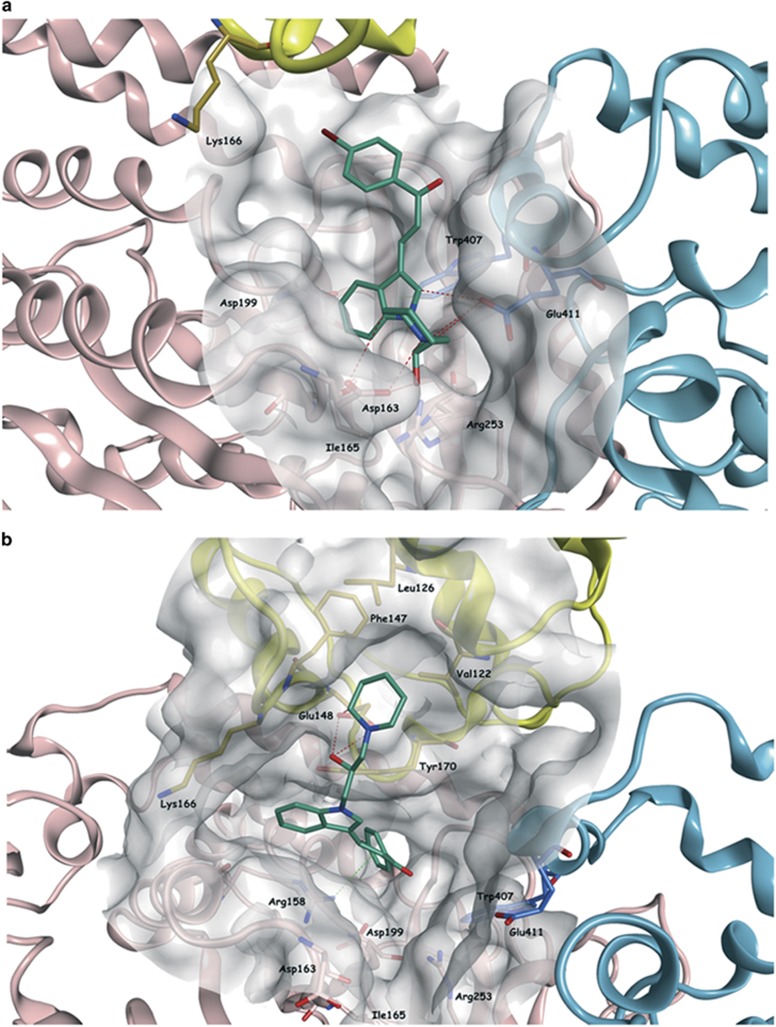
SM15 binding conformation after MD simulation. (**a**) Predicted binding mode of SM15 (carbon atoms in emerald) in the selected binding area between the two tubulin subunits. The molecule is interacting with the surrounding residues (for example Asp163, Trp407 and Glu411) placing the indole-bromophenyl–propenone group deep in the pocket. (**b**) After 100 ns MD simulation, the piperidine ring of SM15 moved toward Hec1 inserting itself in a small hydrophobic pocket within the calponin homology domain formed by Val122, Leu126, Phe147 and Tyr170 (carbon atoms in gold). The rest of the molecule, especially the bromophenyl–propenone part, remained between the two tubulin subunits. Interactions of SM15 with Hec1 and β-tubulin are represented as dashed red and green lines, respectively.

To gain insights into the molecular mechanism mediating the interaction of SM15 with its targets, we investigated the modification exerted by SM15 on MTs and KT–MT attachments at the molecular level by carrying out a molecular dynamics (MD) simulation of the tubulin dimer–Hec1 complex in the presence of SM15. The predicted binding mode of SM15 is illustrated in Figure 7a and shows that the molecule occupies the selected binding area between the two tubulin subunits, interacting with the surrounding residues (for example, Asp163, Trp407 and Glu411) and placing the indole-bromophenyl–propenone group deep in the pocket. This best binding pose obtained for SM15 after docking at the isolated tubulin dimer interface was used as starting point for the simulation. After 35 ns of simulation, part of the compound gradually moved from the initial position on the pocket between the α- and β-tubulin interface toward the Hec1 protein. After 45 ns, SM15 reached a binding conformation, inserted between the two tubulin subunits and Hec1, that was maintained until the end of the simulation. Particularly, the piperidine ring moved toward Hec1 inserting itself in a small hydrophobic pocket formed by Val122, Leu126, Phe147 and Tyr170. The rest of the molecule, especially the bromophenyl–propenone part, remained between the two tubulin subunits, maintaining some interactions with the tubulin dimer (Figure 7b). Altogether, these data indicate that the molecule can bind the MT surface independently from Hec1 and acts as a stabilizer of both the MT and the MT–KT interaction.

## Discussion

KTs represent an interesting anticancer drug target owing to their fundamental role in mitosis. In an effort to find SMs acting at KTs we carried out a virtual screening study targeting the interaction surface area between Hec1 and MTs. A new interesting and well-defined pocket at the interface between the two tubulin subunits was identified and its vicinity to the tubulin-Hec1 contact area made this pocket a potential target for our *in silico* studies. The virtual screening process led to the identification of two new SMs (SM15 and SM17) with elevated anti-proliferative, pro-apoptotic and anti-mitotic activities. The molecular interaction of SM15 to MTs had been further clarified. Docking studies on SM15 showed that the compound could potentially bind at the tubulin dimerization interface and could interact with amino acids from both α- and β-tubulin, acting as potential bridge (stabilizer) between the two tubulin subunits. These findings are in line with the SPR results, in which SM15 was able to bind directly and with high affinity to MTs and (to a lesser extent) to tubulin. Our results also show that SM15 promotes chromosome mis-segregation by inhibiting the correction of faulty KT–MT attachments through the stabilization of the KT–MT interaction. MD simulation studies showed how SM15, after potentially binding at the tubulin dimer interface, could interact with Hec1 forming a potential bridge between Hec1 and tubulin, thereby stabilizing the KT–MT interaction. Furthermore, the molecular modeling suggests that SM15 can interact with tubulin also in the absence of Hec1, stabilizing MTs and does not affect the independent binding of Hec1 to tubulin. Live cell imaging demonstrates that cell death intervenes both from mitosis and from interphase and that this phenotype is associated with hyper-stable mitotic and interphase MTs. Overall, these experimental data, supported by the molecular modeling results, suggest that SM15 acts specifically toward the MTs, stabilizing MT dynamics in mitosis and interphase. Thus, we can conclude that the virtual screening study designed to identify novel molecules able to inhibit/modify the MT–Hec1 interaction led to the identification of a novel tubulin stabilizer drug. The tested molecule has the unique characteristic of interacting with the external surface of the MT cylinder, at odds with known MT stabilizing agents that interact at specific locations within the MT lumen.^28^ Apart from a recent work that evidenced a binding site for paclitaxel at the outer MT surface before the drug reaches the internal lumen site,^29^ several interaction sites have been mapped for MT stabilizing agents, but all reside in the internal lumen.^28^ The interaction of SM15 on external surface of the MTs could provide an explanation for its effects outside mitosis, since its binding to MTs could efficiently affect already assembled interphase MTs. This conclusion is supported by the modeling studies that delineate the binding site of SM15 on the external interface between the tubulin chains. In addition, SM15 could stabilize the KT–MT interaction by bridging the tubulin dimer and Hec1.

In conclusion, starting from a rational approach, we identified a new compound, SM15, which present a dual mechanism: MT stabilizer agent and stabilizer of KT–MT interactions. Both these properties give to SM15 the potentialities to emerge as a novel powerful anticancer agent, promoting chromosome mis-segregation by stabilizing faulty KT–MT attachments. In addition to this mechanism, stabilization of MT dynamics in interphase by SM15 may represent an important feature of its anticancer activity, by promoting massive apoptosis of malignant cells, independently of mitosis.

## Materials and methods

### Molecular modeling

All molecular docking studies were performed on a Viglen Genie Intel Core i7-3770 vPro CPU@ 3.40 GHz × 8 running Ubuntu 14.04. Molecular Operating Environment (MOE) 2015.10^30^ and Maestro (Schrödinger Release 2016-1)^31^ were used as modeling softwares. The cryo-electron microscopy density map and the Ndc80 docking model coordinates were downloaded from the PDB data bank (PDB code 3IZ0).^16^ The tubulin dimer (chains A and B) together with the Ndc80-SPC25 chimera protein (Hec1-containing chain C) were extracted from the protein model. The complex was preprocessed using the Schrödinger Protein Preparation Wizard by assigning bond orders, adding hydrogens and performing a restrained energy minimization of the added hydrogens using the OPLS_2005 force field. The library of commercially available compounds was downloaded from SPECS website (www.specs.net) in a sdf format and prepared using the Maestro LigPrep tool by energy minimizing the structures (OPLS_2005 force field), generating possible ionization states at pH 7±2, generating tautomers and low-energy ring conformers. After removing the Ndc80-SPC25 chimera protein, a 12Å docking grid (inner-box 10Å and outer-box 22Å) was prepared using as centroid the area defined by Glu411 and Trp407 (α-tubulin chain) and Asp199, Ile165, Asp163 and Arg253 (β-tubulin chain) in the isolated tubulin dimer structure. Molecular docking of the prepared SPECS library was performed using Glide HTVS. The best 5000 ranked compounds obtained were docked using the more accurate Glide SP precision setting 1 as output pose per input structure. The output poses were then finally docked with Glide XP scoring function and the output database saved as mol2 file. The best 500 compounds according to the Glide XP results were visually inspected for their ability to bind the active site and 15 derivatives were selected and purchased.

MD simulations were performed on Supermicro Intel Xeon CPU ES-46200 @ 2.20 GHz × 12 running Ubuntu 14.04 using the Desmond package for MD simulation: OPLS-AA force field in explicit solvent, employing the TIP3 water model was used. The initial coordinates for the MD simulation were taken from the best docking result for compound SM15 at the tubulin dimer interface. A cubic water box was used for the solvation of the system, ensuring a buffer distance of ~10 Å between each box side and the complex atoms. The system was neutralized adding 27 sodium counter ions. The system was minimized and pre-equilibrated using the default relaxation routine implemented in Desmond. A 100 ns MD simulation was performed, during which the equations of motion were integrated using a 2 fs time step in the NPT ensemble, with temperature (300 K) and pressure (1atm) constant. All other parameters were set using the Desmond default values. Data were collected every 40ps (energy) and every 160 ps (trajectory). Visualization of protein-ligand complex and MD trajectory analysis were carried out using Maestro. The MD simulation was performed in presence of Hec1 protein isolated from the Ndc80-SPC25 chimera protein (124 amino acids from Met79 to Ser202).

### Cell culture and viability assays

HeLa cells, MDA-MB-231 breast cancer cells (originally purchased from ATCC) and U2OS cells stably expressing H2B-GFP and RFP-α-tubulin (a kind gift of L Lanzetti, Institute for Cancer Research at Candiolo, Italy) were grown in DMEM medium supplemented with 10% fetal bovine serum, 1% l-glutamine and antibiotics. MCF10A cells were cultured in DMEM/F12 medium supplemented with 5% horse serum, 10 μg/ml insulin, 0.5 μg/ml hydrocortisone and 20 ng/ml epidermal growth factor. Cells were grown at 37 °C in a humidified atmosphere containing 5% CO_2_ and routinely tested for mycoplasm contamination. All SMs were stored as 10 mM stock solutions in DMSO at −20 °C and added after serial dilutions in order not to exceed 1% DMSO in medium. A total of 6 × 10^5^ HeLa cells were seeded in 25 cm^2^ flasks and exposed 24 h later to different concentrations of SMs. Cells were harvested 24 h later to obtain IC_50_ values or after 24, 48, 72 h from SM addition in time course experiments. Cell counting was performed by a Z1 Coulter Counter (Beckman Coulter, Brea, CA, USA) and cell viability was assessed by trypan blue exclusion. Cell cycle progression and apoptosis were evaluated on the same samples by flow cytometry on an Epics XL apparatus (Beckman Coulter) using propidium iodide staining. Ten thousand events were collected from each sample.

### SPR experiments

SPR experiments were carried out using a SensiQ Pioneer system. The sensor chip (COOH5) was activated chemically by a 35 μl injection of a 1:1 mixture of *N*-ethyl-*N*’-3-(diethylaminopropyl)carbodiimide (200 mM) and *N*-hydroxysuccinimide (50 mM) at a flow rate of 5 μl/min. Ligands, that is, tubulin (3900 RU) and assembled MTs (6050 RU), were immobilized on activated sensor chips via amine coupling as previously reported:^32^ immobilizations were carried out in 10 mM sodium acetate at pH 4.0; flow cell Fc2 was left empty and used as a reference surface. The remaining ester groups were blocked by injecting 1 M ethanolamine hydrochloride (35 μl). SM15 was dissolved in DMSO to a final concentration of 10 mM, and then diluted 1:50 in buffer A (10 mM Hepes pH 7.4, 150 mM NaCl+0.05% surfactant P20+2 mM MgCl_2_). Further dilutions of SM15 were carried out in running buffer (buffer A+2% DMSO); each sample was injected in duplicate on the sensor chip for 240 s (contact time) followed by a dissociation of 1000 s, at a flow rate of 30 μl/min. Regeneration procedures included two long (1000 s and 300 s) injections of buffer, separated by a 30 s injection of 10 mM glycine pH 1.5. The sensorgrams were analyzed using the SensiQ Qdat program.

### Western blotting

Western blot experiments were performed as previously described.^33^ In brief, cells were lysed in RIPA buffer and 40 μg of total proteins were resolved in 4–12% gradient gels by SDS-PAGE. Proteins were transferred onto nitrocellulose membranes and incubated with rabbit anti-poly ADP-ribose polymerase (11835238001, Roche Diagnostics GmbH, Mannheim, DE, USA), active caspase 3 (559565, BD Biosciences, St Jose, CA, USA) and goat anti-actin (Sc1616, Santa Cruz Biotechnology, Santa Cruz, CA, USA) antibodies. Protein lysates from tumors were probed with poly ADP-ribose polymerase cleavage antibody (AB3565, Millipore, Billerica, MA, USA) and HSP72/73 antibody (HSP01, Millipore). The membranes were subsequently probed with the appropriate horseradish peroxidase-conjugated secondary antibodies (Santa Cruz Biotechnology) and antigens on the membrane were revealed by enhanced chemiluminescence (RPN2209, GE Healthcare, Little Chalfont, UK).

### Immunofluorescence microscopy and analysis

Cells were rinsed in PHEM (60 mM PIPES, 25 mM Hepes, 10 mM EGTA, 2 mM MgCl_2_) buffer, fixed in 3.7% formaldehyde in PHEM, permeabilized in 0.3% Triton X-100 and post-fixed in cold methanol. Coverslips were processed for immunofluorescence using the following antibodies: anti-α- or γ-tubulin (T6199, T3559, Sigma-Aldrich, St Louis, MO, USA), anti-KT serum (CREST, Antibodies Inc., Davis, CA, USA), anti-Hec1 (GTX70268, Genetex, Irvine, CA, USA). DNA was counterstained with 0.05 μg/ml 4,6-diamidino-2-phenylindole (DAPI, Sigma-Aldrich). Preparations were examined under an Olympus AX70 microscope using a 100 × /1.35 NA objective. Images were acquired using a TCH-1.4ICE camera (Tucsen, Fujian, China) controlled by ISCapture and processed by Photoshop CS. Measurements of fluorescence intensity over the cell area were obtained from images acquired under identical exposure settings using NIH ImageJ 1.3 software.

### Live cell microscopy

A total of 3 × 10^4^ HeLa or U2OS cells/well were seeded in four-well microslides (80426, Ibidi, Martinsried, DE) and time-lapse observation started 24 h later, when 5 μM SM15 was added to the medium. Cells were recorded under an Eclipse Ti inverted microscope (Nikon, Tokyo, Japan), using a Plan Fluor 40 × /0.6 NA or 60 × /0.7 NA objective (Nikon) for DIC and fluorescence, respectively; during the whole observation cells were kept in a microscope stage incubator (Basic WJ, Okolab, Naples, Italy) at 37 °C and 5% CO_2_. Images were acquired over 24 h at 3 min for phase contrast or DIC and 30 min for fluorescence. Videos and still images were processed using NIS-Elements AR 3.2.

### Tumor xenografts and TUNEL in nude mice

HeLa cells in exponential growth phase were harvested from the culture, washed and resuspended in PBS and 4 × 10^6^ cells were subcutaneously injected into 6–8-week-old male CD-1 nude (nu/nu) mice. After the appearance of palpable tumors, animals were allocated into treatment groups (*n*=8/group) on the basis of their tumor volume to obtain two similar groups, that subsequently received either no treatment or SM15. Two different experiments were performed. Animals were intraperitoneally treated with SM15 (10 mg/kg) sonicated and dissolved in 1% carboxymethyl cellulose for five consecutive days for three weeks starting from tumor palpability. Diet consumption, body-weight loss, and postural and behavioral changes were monitored daily. Animals were sacrificed 25 days after cell injection, tumors were removed and proteins extracted. We applied the Shapiro–Wilk test to evaluate the normality of the weight distribution of the treated mice and controls. Both distributions, at the two time points, were not normally distributed. Thus, in order to explore potential differences between the two treatment groups we used the non-parametric Mann–Whitney test. Results were considered to be statistically significant if *P*<0.05. Mice were purchased from Charles River Laboratories (Calco, Italy). All procedures involving animals and their care were authorized and certified by D.lgs 26/2014 (816/2015-PR del 11/08/2015) of the Italian Health Ministry. The immunohistochemical detection of apoptosis was performed by TUNEL assay using a commercial kit (*In Situ* Cell Death Detection Kit, POD, Roche). The assay was performed according to the manufacturer's instructions. For each tumor, three different 5 μm frozen sections were analyzed and examined by light microscopy. Sections were scanned at × 200 magnification. Apoptosis of tumor cells was counted in four high-power fields (× 400 magnification) per section.

### Statistical analysis

All data are presented as mean±s.e.m. Significance of differences between experimental variables was determined by unpaired two-tailed Student’s *t*-test unless otherwise stated. A *P*-value of >0.05 was considered significant. Statistical analyses were performed using the GraphPad Prism program. The sample sizes were determined by power analysis, based on variation shown in our previous experiments.^12^ No data were excluded from any analysis. Evaluation of immunofluorescence and TUNEL samples was performed independently and in blinded manner by two investigators.
