# Supplementary material for: Small molecules targeted to the microtubule–Hec1 interaction inhibit cancer cell growth through microtubule stabilization
Source: Oncogene. 2017 Sep 18;37(2):231–40. doi: 10.1038/onc.2017.320 (PMC5770598; doi:10.1038/onc.2017.320)
Supplement: Supplementary Figures [file onc2017320x1.pdf]

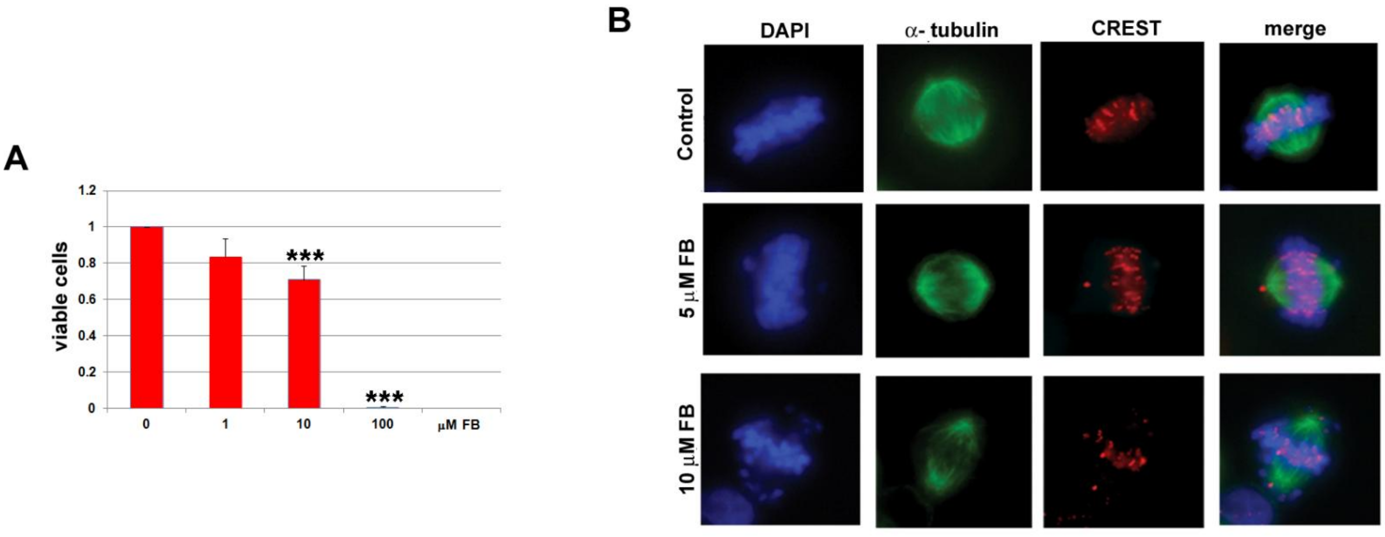

Supplementary Figure 1. Characterization of the hit compound FB, identified in the virtual screening for molecules interacting at the MT-Hec1 interface. (A) Inhibition of cell viability after 24 hr treatments with FB in HeLa cells. Data are means ± SEM of 2 independent experiments and are expressed as fold difference over the control value. $P < 0.001$ Student's t-test. (B) Induction of chromosome congression defects, as visualized by the presence of unaligned polar chromosomes in HeLa cells treated for 3 hrs with 5 or 10 µM FB. Kinetochores (red) and mitotic spindles (green) are detected by CREST and α tubulin immunostaining. Chromosomes are identified by DAPI staining (blue).

ID	STRUCTURE	IC50 (μM)	ID	STRUCTURE	IC50 (μM)
FB	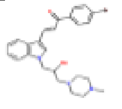	20.18	10	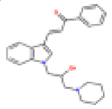	49.77
1	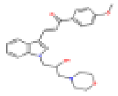	72.28	11	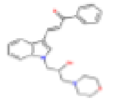	17.22
2	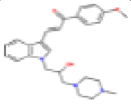	51.76	12	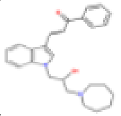	21.23
3	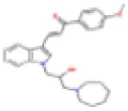	51.29	13	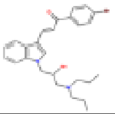	>100
4	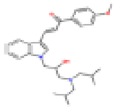	>100	14	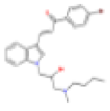	6.11
5	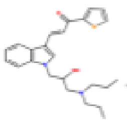	20.08	15	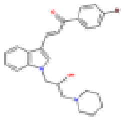	6.00
6	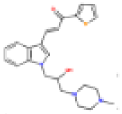	20.14	16	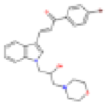	>100
7	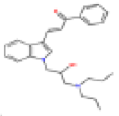	20.70	17	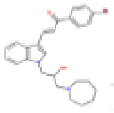	15.38
8	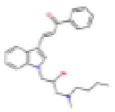	28.84	18	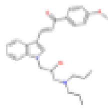	31.70
9	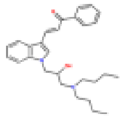	22.70	19	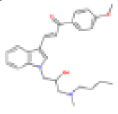	27.23

Supplementary Figure 2. Cytotoxicity of MT-Hec1 interacting small molecules. Calculated IC 50 for cell viability in HeLa cells treated for 24 hrs with the hit compound FB or 19 analogues of FB.in the dose range 1-100 μM. The structure of each small molecule is also shown.

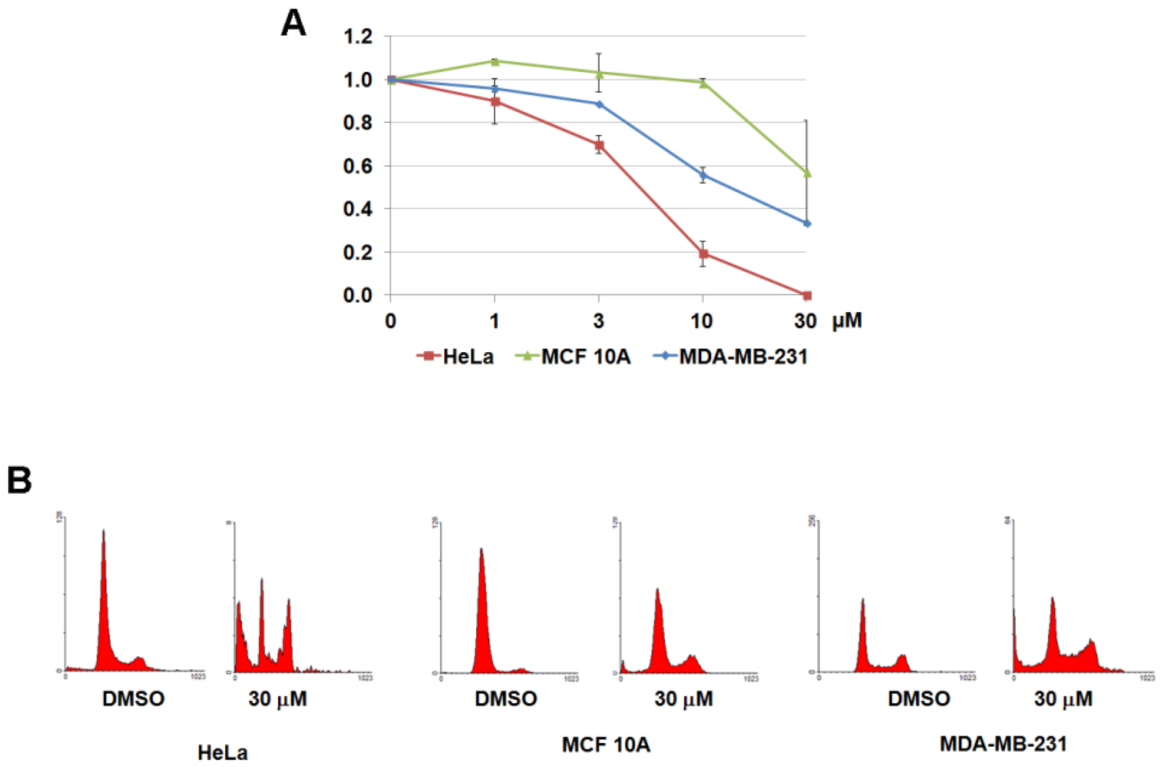

Supplementary Figure 3. Increased sensitivity of tumor cells to the cell killing ability of SM 15. (A) Inhibition of cell viability after 24 hr treatments of HeLa, MCF 10A and MDA-MB-231 cells with different concentrations of SM 15. Data are means ± SEM of 2-3 independent experiments and are expressed as fold difference over the respective control values. (B) Flow cytometric histograms of DNA content (X axis) and number of events (Y axis) in cells treated with 1% DMSO or after 24 hr exposure to 30 μM SM 15 in HeLa, MCF 10A and MDA-MB-231 cells.

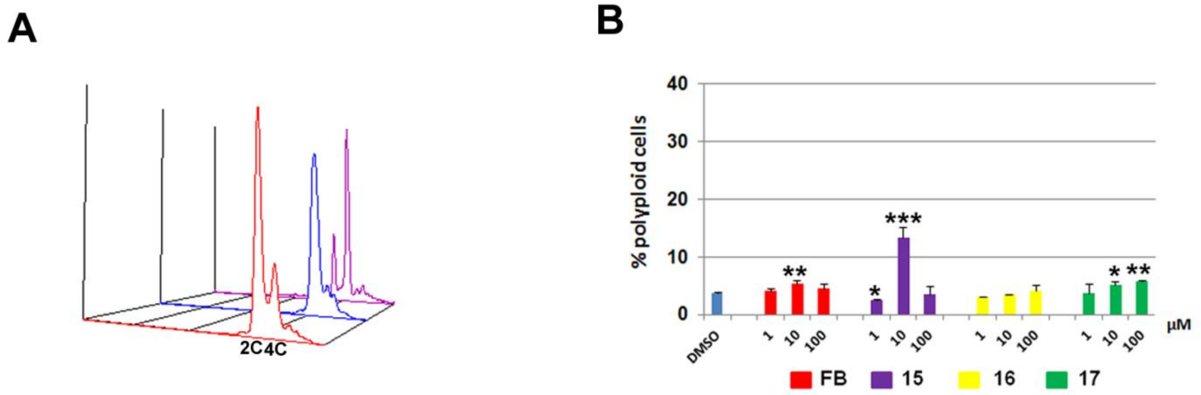

Supplementary Figure 4. Induction of polyploidy in Hela cells. (A) Representative flow cytometric histograms of DNA content following 24 hr exposure to 1% DMSO 10 µM of FB, SM 15 or. X axis = DNA content (logarithmic scale), Y axis= number of events. (B) Quantitative analysis of the percentage of cells showing a DNA content > 4 C after 24 hr exposure to FB, SM 15, SM 16 and SM 17. Data are means ± SEM of 2-4 independent experiments.

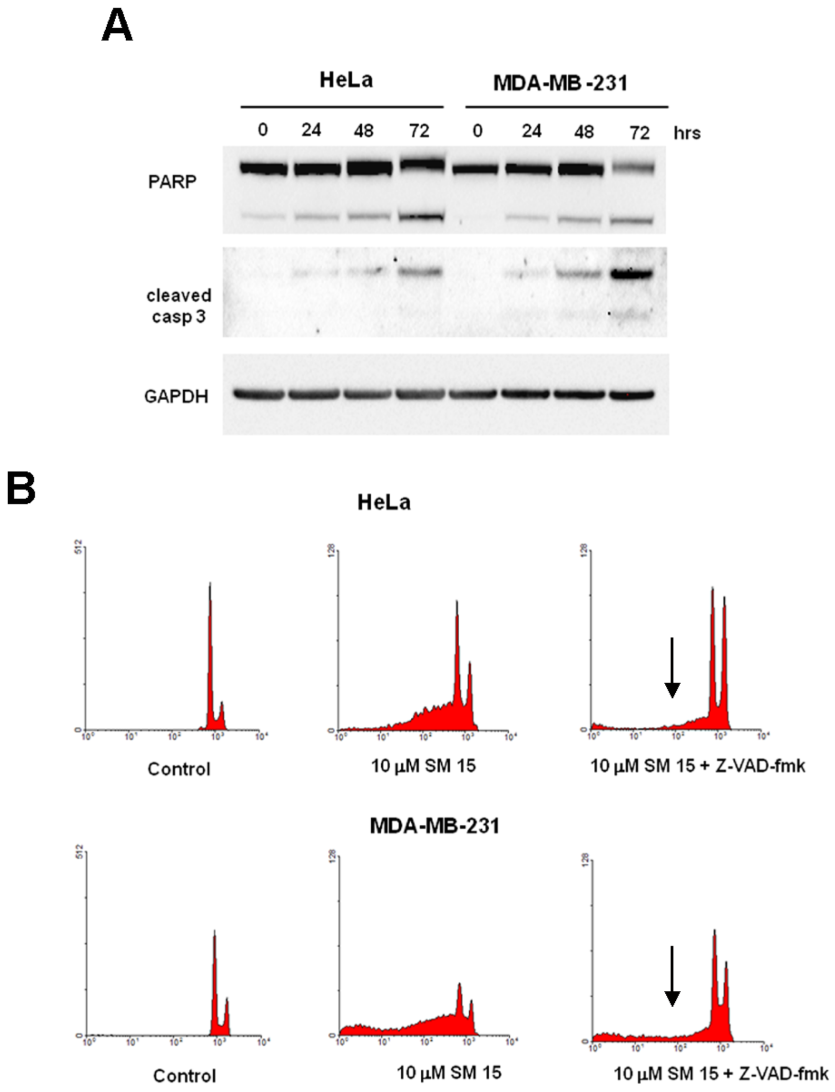

Supplementary Figure 5. Apoptosis induction in HeLa and MDA-MB-231 cells. A) Western blot analysis of PARP cleavage and cleaved caspase 3 after different exposure times to 10 μM SM15 in HeLa and MDA-MB-231 cells. GAPDH is shown as loading control. B) Cytofluorimetric analysis of DNA content in HeLa and MDA-MB-231 cells treated with 10 μM SM 15 for 48 hrs, simultaneously treated with10 μM SM 15 for 48 hrs and 25 μM Z-VAD-fmk or left untreated. The arrows point to the fraction of cells showing an hypodiploid DNA content that is severely reduced in the combined treatment in both cell lines.

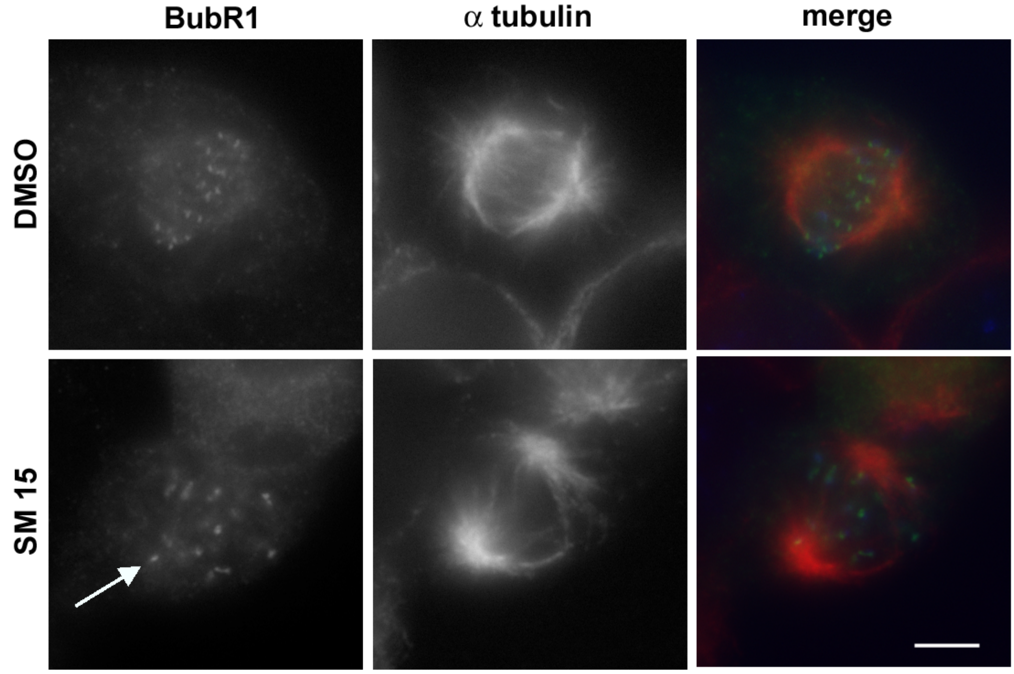

Supplementary Figure 6. SAC activation in SM 15 treated HeLa cells. Representative images of HeLa cells treated with 10 μM SM 15 or treated with 1% DMSO for 3 hrs and immunostained using an anti α-tubulin antibody (Sigma-Aldrich) and an anti-BubR1 antibody (A300-386A, Bethyl). Tubulin is visualized in red and BubR1 in green. Bar = 5 μm. In SM 15 treated cells, kinetochores do not align to the metaphase plate and accumulate BubR1. Arrow points to a kinetochore localized at spindle poles with higher BubR1 fluorescence intensity.
